# Bis(2-hy­droxy­ethanaminium) terephthalate

**DOI:** 10.1107/S1600536812000293

**Published:** 2012-01-11

**Authors:** Yu Jin

**Affiliations:** a Ordered Matter Science Research Center, Southeast University, Nanjing 211189, People’s Republic of China

## Abstract

The asymmetric unit of the title salt, 2C_2_H_8_NO^+^·C_8_H_4_O_4_^2−^, comprises one crystallographically independent 2-hy­droxy­ethanaminium cation and one half terephthalate anion. In the crystal, hydrogen bonds involving the hy­droxy and ammonium groups of the cations and the carboxyl­ate O atoms of the terephthalate anions result in the formation of a three-dimensional network structure.

## Related literature

For compounds containing the terephthalate anion, see: Zhang *et al.* (2005 ▶); Smith & Wermuth (2010 ▶); Karpova *et al.* (2004 ▶). For their physical properties, see: Ye *et al.* (2006 ▶); Zhang *et al.* (2008 ▶, 2009 ▶, 2010 ▶); Fu *et al.* (2009 ▶); Wu *et al.* (2011 ▶).
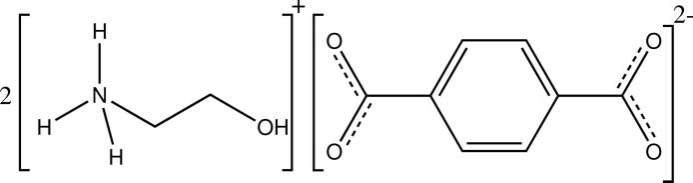


## Experimental

### 

#### Crystal data

2C_2_H_8_NO^+^·C_8_H_4_O_4_^2−^
*M* *_r_* = 288.30
Monoclinic, 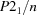

*a* = 9.3578 (19) Å
*b* = 7.8579 (16) Å
*c* = 9.844 (2) Å
β = 110.53 (3)°
*V* = 677.9 (2) Å^3^
*Z* = 2
Mo *K*α radiation
μ = 0.11 mm^−1^
*T* = 293 K
0.3 × 0.3 × 0.2 mm

#### Data collection

Rigaku Mercury CCD diffractometer
Absorption correction: multi-scan (*CrystalClear*; Rigaku, 2005 ▶) *T*_min_ = 0.489, *T*_max_ = 1.000
6639 measured reflections
1558 independent reflections
1270 reflections with *I* > 2σ(*I*)
*R*_int_ = 0.068

#### Refinement

*R*[*F* ^2^ > 2σ(*F* ^2^)] = 0.044
*wR*(*F* ^2^) = 0.108
*S* = 1.05
1558 reflections
92 parameters
H-atom parameters constrained
Δρ_max_ = 0.29 e Å^−3^
Δρ_min_ = −0.21 e Å^−3^

### 

Data collection: *CrystalClear* (Rigaku, 2005 ▶); cell refinement: *CrystalClear*; data reduction: *CrystalClear*; program(s) used to solve structure: *SHELXS97* (Sheldrick, 2008 ▶); program(s) used to refine structure: *SHELXL97* (Sheldrick, 2008 ▶); molecular graphics: *SHELXTL* (Sheldrick, 2008 ▶); software used to prepare material for publication: *SHELXL97*.

## Supplementary Material

Crystal structure: contains datablock(s) I, global. DOI: 10.1107/S1600536812000293/bx2389sup1.cif

Structure factors: contains datablock(s) I. DOI: 10.1107/S1600536812000293/bx2389Isup2.hkl

Supplementary material file. DOI: 10.1107/S1600536812000293/bx2389Isup3.cml

Additional supplementary materials:  crystallographic information; 3D view; checkCIF report

## Figures and Tables

**Table 1:** Hydrogen-bond geometry (Å, °)

*D*—H⋯*A*	*D*—H	H⋯*A*	*D*⋯*A*	*D*—H⋯*A*
O1—H1*A*⋯O3^i^	0.82	1.92	2.7373 (15)	179
N1—H1*F*⋯O3^ii^	0.89	2.03	2.8995 (16)	164
N1—H1*C*⋯O3^iii^	0.89	2.15	2.9725 (16)	154
N1—H1*B*⋯O2^iv^	0.89	1.80	2.6792 (15)	169

Symmetry codes: (i) 
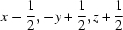
; (ii) 
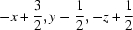
; (iii) 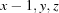; (iv) 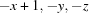.

**Fig. 1.**
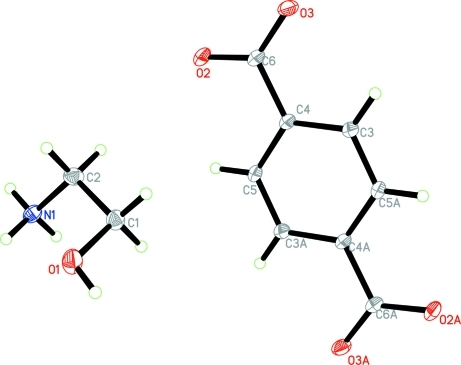
Crystal structure of the title compound with labelling and displacement ellipsoids drawn at the 30% probability level.Symmetry code (A): 2-x,-y,1-z

**Fig. 2.**
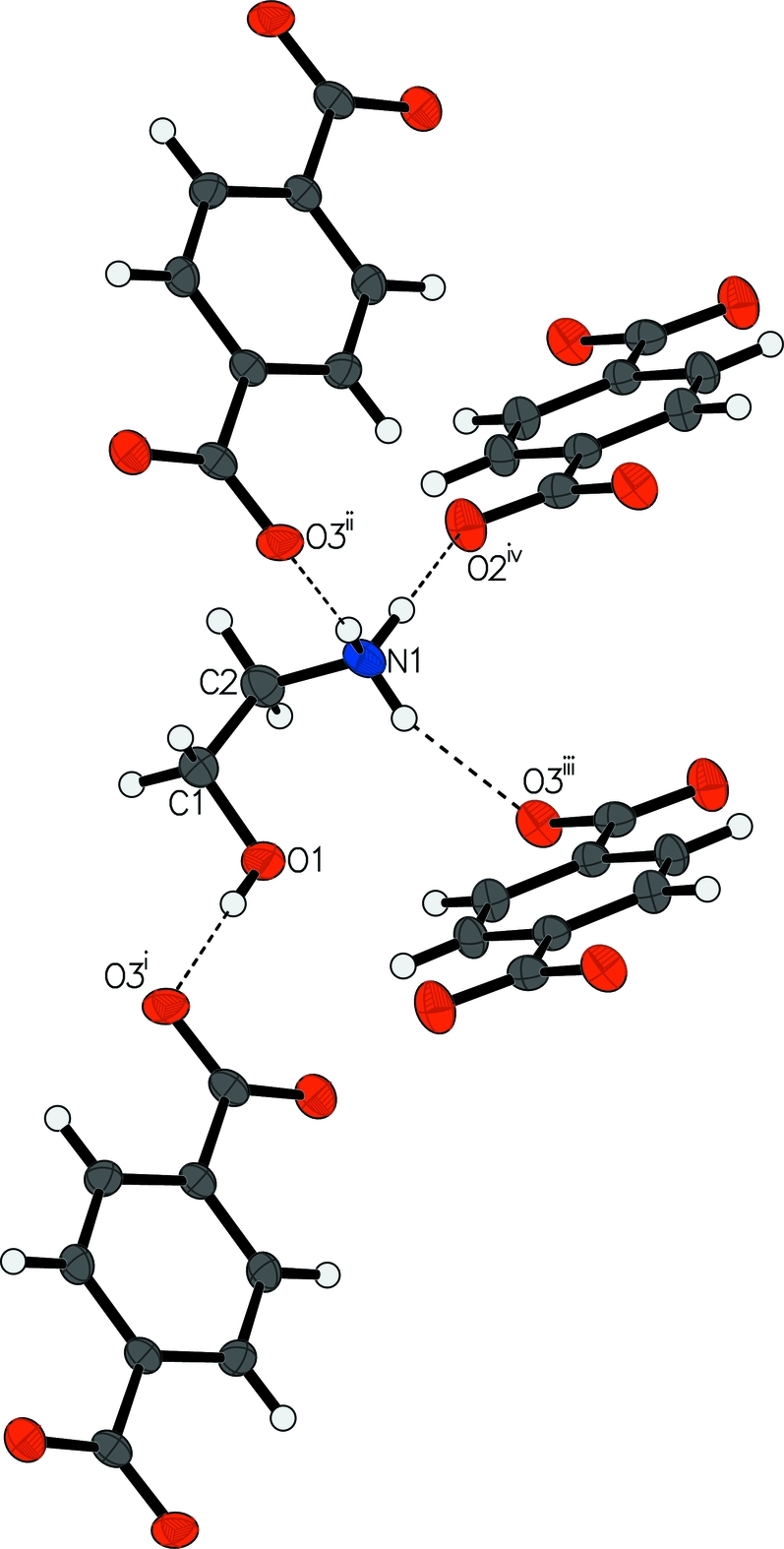
A view of the association between ions in bis (2-hydroxyethanaminium) terephthalate, showing the hydrogen bonds interactions. Symmetry code: (i) x-1/2, -y+1/2, z+1/2; (ii) -x+3/2, y-1/2, -z+1/2; (iii) x-1, y, z; (iv) -x+1, -y, -z.

## supplementary crystallographic information

### Comment

Several crystal structures containing terephthalate anion have been reported previously (Zhang *et al.*, 2005; Smith & Wermuth, 2010; Karpova *et al.*, 2004) as well as their physical properties (Zhang *et al.*, 2010; Zhang *et al.*, 2008; Wu *et al.*, 2011). We report here the crystal structure of the title compound, Fig.1. The asymmetric unit of the title salt, 2(C_2_H_8_NO)^+^. (C_8_H_4_O_4_)^2-^ comprises one crystallographically independent 2-hydroxyethanaminium cation and one-half-terephthalate anion. In the crystal structure, hydrogen bonds involving the hydroxy and ammonium groups connect the carboxyl O atoms of the terephthalate anion into a three-dimensional network structure, Figure 2, Table1.

### Experimental

The title compound was synthetized from a mixture of NH_2_(CH_2_)_2_OH (122.16 mg, 2.00 mmol), C_8_H_6_O_4_ (166.13 mg, 1.00 mmol), and distilled water (10 mL), which was stirred a few minutes at room temperature, giving a clear transparent solution. After evaporation for a few days, block colorless crystals suitable for X-ray diffraction were obtained in about 77% yield, which were filtered and washed with distilled water.

### Refinement

H atoms bound to carbon and nitrogen were placed at idealized positions [C—H = 0.93 to 0.97 Å, N—H = 0.89 Å and O—H = 0.82 Å] and allowed to ride on their parent atoms with *U*_iso_ fixed at 1.2 *U*_eq_(C,*N*).

### Crystal data

**Table:**

2C_2_H_8_NO^+^·C_8_H_4_O_4_^2^^−^	*F*(000) = 308
*M**_r_* = 288.30	*D*_x_ = 1.412 Mg m^−^^3^
Monoclinic, *P*2_1_/*n*	Mo *K*α radiation, λ = 0.71073 Å
Hall symbol: -P 2yn	Cell parameters from 3450 reflections
*a* = 9.3578 (19) Å	θ = 6.2–55.3°
*b* = 7.8579 (16) Å	µ = 0.11 mm^−^^1^
*c* = 9.844 (2) Å	*T* = 293 K
β = 110.53 (3)°	Block, colorless
*V* = 677.9 (2) Å^3^	0.3 × 0.3 × 0.2 mm
*Z* = 2	

### Data collection

**Table:**

Rigaku Mercury CCD diffractometer	1558 independent reflections
Radiation source: fine-focus sealed tube	1270 reflections with *I* > 2σ(*I*)
graphite	*R*_int_ = 0.068
ω scans	θ_max_ = 27.5°, θ_min_ = 3.4°
Absorption correction: multi-scan (*CrystalClear*; Rigaku, 2005)	*h* = −12→12
*T*_min_ = 0.489, *T*_max_ = 1.000	*k* = −10→10
6639 measured reflections	*l* = −12→12

### Refinement

**Table:**

Refinement on *F*^2^	Secondary atom site location: difference Fourier map
Least-squares matrix: full	Hydrogen site location: inferred from neighbouring sites
*R*[*F*^2^ > 2σ(*F*^2^)] = 0.044	H-atom parameters constrained
*wR*(*F*^2^) = 0.108	*w* = 1/[σ^2^(*F*_o_^2^) + (0.0417*P*)^2^ + 0.0687*P*] where *P* = (*F*_o_^2^ + 2*F*_c_^2^)/3
*S* = 1.05	(Δ/σ)_max_ < 0.001
1558 reflections	Δρ_max_ = 0.29 e Å^−^^3^
92 parameters	Δρ_min_ = −0.20 e Å^−^^3^
0 restraints	Extinction correction: *SHELXL97* (Sheldrick, 2008), Fc^*^=kFc[1+0.001xFc^2^λ^3^/sin(2θ)]^-1/4^
Primary atom site location: structure-invariant direct methods	Extinction coefficient: 0.330 (17)

### Special details

**Table:**

Geometry. All e.s.d.'s (except the e.s.d. in the dihedral angle between two l.s. planes) are estimated using the full covariance matrix. The cell e.s.d.'s are taken into account individually in the estimation of e.s.d.'s in distances, angles and torsion angles; correlations between e.s.d.'s in cell parameters are only used when they are defined by crystal symmetry. An approximate (isotropic) treatment of cell e.s.d.'s is used for estimating e.s.d.'s involving l.s. planes.
Refinement. Refinement of *F*^2^ against ALL reflections. The weighted *R*-factor *wR* and goodness of fit *S* are based on *F*^2^, conventional *R*-factors *R* are based on *F*, with *F* set to zero for negative *F*^2^. The threshold expression of *F*^2^ > σ(*F*^2^) is used only for calculating *R*-factors(gt) *etc*. and is not relevant to the choice of reflections for refinement. *R*-factors based on *F*^2^ are statistically about twice as large as those based on *F*, and *R*- factors based on ALL data will be even larger.

### Fractional atomic coordinates and isotropic or equivalent isotropic displacement parameters (Å^2^)

**Table:**

	*x*	*y*	*z*	*U*_iso_*/*U*_eq_	
C1	0.52569 (16)	0.15091 (18)	0.33959 (14)	0.0269 (3)	
H1D	0.5597	0.0514	0.4007	0.032*	
H1E	0.6141	0.2202	0.3476	0.032*	
C2	0.44639 (17)	0.09872 (18)	0.18647 (14)	0.0280 (4)	
H2A	0.4138	0.1992	0.1262	0.034*	
H2B	0.5164	0.0356	0.1525	0.034*	
C3	1.10759 (16)	0.09307 (16)	0.46571 (14)	0.0229 (3)	
H3A	1.1809	0.1552	0.4432	0.028*	
C4	0.97258 (15)	0.04996 (15)	0.35739 (12)	0.0196 (3)	
C5	0.86558 (16)	−0.04471 (16)	0.39320 (14)	0.0226 (3)	
H5A	0.7747	−0.0758	0.3209	0.027*	
C6	0.93996 (16)	0.10265 (16)	0.20297 (13)	0.0216 (3)	
N1	0.31233 (13)	−0.00831 (14)	0.17323 (11)	0.0251 (3)	
H1B	0.2661	−0.0380	0.0809	0.038*	
H1C	0.2477	0.0500	0.2036	0.038*	
H1F	0.3425	−0.1014	0.2272	0.038*	
O1	0.42127 (12)	0.24468 (13)	0.38314 (11)	0.0356 (3)	
H1A	0.4584	0.2642	0.4704	0.053*	
O2	0.81514 (12)	0.05985 (13)	0.11320 (10)	0.0318 (3)	
O3	1.04092 (11)	0.18757 (12)	0.17413 (10)	0.0281 (3)	

### Atomic displacement parameters (Å^2^)

**Table:**

	*U*^11^	*U*^22^	*U*^33^	*U*^12^	*U*^13^	*U*^23^
C1	0.0241 (8)	0.0296 (7)	0.0252 (8)	−0.0011 (6)	0.0065 (6)	−0.0043 (5)
C2	0.0305 (8)	0.0314 (7)	0.0234 (8)	−0.0052 (6)	0.0111 (6)	−0.0034 (5)
C3	0.0221 (7)	0.0273 (7)	0.0189 (7)	−0.0028 (5)	0.0065 (6)	0.0027 (5)
C4	0.0222 (7)	0.0210 (6)	0.0144 (7)	0.0021 (5)	0.0047 (6)	0.0004 (5)
C5	0.0192 (7)	0.0281 (7)	0.0166 (7)	−0.0013 (5)	0.0016 (6)	0.0002 (5)
C6	0.0267 (8)	0.0214 (6)	0.0153 (7)	0.0039 (5)	0.0057 (6)	0.0009 (5)
N1	0.0293 (7)	0.0247 (6)	0.0182 (6)	−0.0022 (5)	0.0045 (5)	−0.0027 (4)
O1	0.0294 (6)	0.0461 (7)	0.0268 (6)	0.0065 (5)	0.0041 (5)	−0.0143 (4)
O2	0.0281 (6)	0.0443 (6)	0.0166 (6)	−0.0036 (4)	0.0000 (5)	0.0042 (4)
O3	0.0340 (6)	0.0312 (6)	0.0187 (5)	−0.0054 (4)	0.0086 (4)	0.0040 (4)

### Geometric parameters (Å, °)

**Table:**

C1—O1	1.4055 (15)	C4—C5	1.3885 (18)
C1—C2	1.485 (2)	C4—C6	1.5000 (17)
C1—H1D	0.9700	C5—C3^i^	1.3755 (18)
C1—H1E	0.9700	C5—H5A	0.9300
C2—N1	1.4776 (17)	C6—O2	1.2387 (18)
C2—H2A	0.9700	C6—O3	1.2676 (15)
C2—H2B	0.9700	N1—H1B	0.8900
C3—C5^i^	1.3755 (18)	N1—H1C	0.8900
C3—C4	1.3795 (19)	N1—H1F	0.8900
C3—H3A	0.9300	O1—H1A	0.8200
			
O1—C1—C2	107.52 (11)	C3—C4—C6	121.42 (12)
O1—C1—H1D	110.2	C5—C4—C6	119.78 (12)
C2—C1—H1D	110.2	C3^i^—C5—C4	120.79 (13)
O1—C1—H1E	110.2	C3^i^—C5—H5A	119.6
C2—C1—H1E	110.2	C4—C5—H5A	119.6
H1D—C1—H1E	108.5	O2—C6—O3	125.00 (12)
N1—C2—C1	110.69 (10)	O2—C6—C4	117.00 (12)
N1—C2—H2A	109.5	O3—C6—C4	118.01 (12)
C1—C2—H2A	109.5	C2—N1—H1B	109.5
N1—C2—H2B	109.5	C2—N1—H1C	109.5
C1—C2—H2B	109.5	H1B—N1—H1C	109.5
H2A—C2—H2B	108.1	C2—N1—H1F	109.5
C5^i^—C3—C4	120.40 (12)	H1B—N1—H1F	109.5
C5^i^—C3—H3A	119.8	H1C—N1—H1F	109.5
C4—C3—H3A	119.8	C1—O1—H1A	109.5
C3—C4—C5	118.81 (11)		

Symmetry codes: (i) −*x*+2, −*y*, −*z*+1.

### Hydrogen-bond geometry (Å, °)

**Table:**

*D*—H···*A*	*D*—H	H···*A*	*D*···*A*	*D*—H···*A*
O1—H1A···O3^ii^	0.82	1.92	2.7373 (15)	179
N1—H1F···O3^iii^	0.89	2.03	2.8995 (16)	164
N1—H1C···O3^iv^	0.89	2.15	2.9725 (16)	154
N1—H1B···O2^v^	0.89	1.80	2.6792 (15)	169

Symmetry codes: (ii) *x*−1/2, −*y*+1/2, *z*+1/2; (iii) −*x*+3/2, *y*−1/2, −*z*+1/2; (iv) *x*−1, *y*, *z*; (v) −*x*+1, −*y*, −*z*.
